# Meander Thin-Film Biosensor Fabrication to Investigate the Influence of Structural Parameters on the Magneto-Impedance Effect

**DOI:** 10.3390/s21196514

**Published:** 2021-09-29

**Authors:** Abkar Sayad, Shah Mukim Uddin, Jianxiong Chan, Efstratios Skafidas, Patrick Kwan

**Affiliations:** 1 Department of Neuroscience, The Alfred Centre, Central Clinical School, Monash University, Melbourne, VIC 3004, Australia; abkar.sayad@monash.edu (A.S.); jianxiong.chan@monash.edu (J.C.); 2 Department of Medicine, The University of Melbourne, Royal Melbourne Hospital, Melbourne, VIC 3050, Australia; suddin1@student.unimelb.edu.au (S.M.U.); sskaf@unimelb.edu.au (E.S.); 3 Department of Electrical and Electronic Engineering, Melbourne School of Engineering, The University of Melbourne, Melbourne, VIC 3010, Australia

**Keywords:** magneto-impedance, thickness, magnetic materials, meander, sensing area, turns

## Abstract

Thin-film magneto-impedance (MI) biosensors have attracted significant attention due to their high sensitivity and easy miniaturization. However, further improvement is required to detect weak biomagnetic signals. Here, we report a meander thin-film biosensor preparation to investigate the fabrication parameters influencing the MI effect. Specifically, we hypothesized that an optimal film thickness and sensing area size ratio could be achieved to obtain a maximum MI ratio. A meander multilayer MI biosensor based on a NiFe/Cu/NiFe thin-film was designed and fabricated into 3-, 6-, and 9-turn models with film thicknesses of 3 µm and 6 µm. The 9-turn biosensor resembled the largest sensing area, while the 3- and 6-turn biosensors were designed with identical sensing areas. The results indicated that the NiFe film thickness of 6 µm with a sensing area size of 14.4 mm^2^ resembling a 9-turn MI biosensor is the optimal ratio yielding the maximum MI ratio of 238%, which is 70% larger than the 3- and 6-turn structures. The 3- and 6-turn MI biosensors exhibited similar characteristics where the MI ratio peaked at a similar value. Our results suggest that the MI ratio can be increased by increasing the sensing area size and film thickness rather than the number of turns. We showed that an optimal film thickness to sensing area size ratio is required to obtain a high MI ratio. Our findings will be useful for designing highly sensitive MI biosensors capable of detecting low biomagnetic signals.

## 1. Introduction

Magnetic sensors have received significant attention in electrical and biomedical research over the past few decades [1,2,3,4]. Technological demands have contributed to the development and existence of the many types of magnetic sensors. When developing these sensors, a combination of parameters must be considered for each specific application. These parameters include the external applied magnetic field, linearity, hysteresis, stability, portability, noise, power consumption, size, and cost [5,6,7,8,9,10,11]. Among these sensors are magneto-impedance (MI) biosensors, which are based on the magneto-impedance effect. The MI effect can be described as the changes that the electric impedance of soft magnetic materials undergo when subjected to an external magnetic field. Soft magnetic materials are considered to be highly sensitive materials and display a series of possible magnetic properties based on sensor structure and geometry [12,13]. Therefore, they have the potential for a wide range of biosensing applications when sensor geometry is optimized. Optimizing sensor geometry such as thickness, lateral dimension, meander turns, and shape enhances the MI response [4]. The MI response magnitude is associated with the sensor sensitivity. Highly sensitive MI biosensors are based on high MI response. Therefore, optimizing the previously mentioned parameters is critical to producing highly sensitive MI biosensors that are capable of detecting low concentrations of biological targets. Moreover, MI biosensors are suitable for the miniaturization that is needed to match the size of biological targets, which enables the sensitive detection of small concentrations compared to other magnetic biosensors such as giant magnetoresistive (GMR) sensors [14,15]. One of the drawbacks of the GMR sensor is the complexity of optimizing its structural parameters. GMR biosensors may have low sensitivity due to the large size difference between their magnetic probes and detection targets [14]. In this regard, GMR sensors have a sensitivity of ~1% Oe, whereas the field sensitivity of a typical MI biosensor can reach a value as high as 500% Oe [16].

Biosensor film thickness significantly affects MI performance. The thickness must be optimized to attain the maximum MI response with regard to magnetic material selection. Recently, the iron–nickle (Fe_20_Ni_80_) permollay (Py) soft magnetic material has shown a significant contribution to the construction of MI biosensors. This material demonstrates large permeability, low magnetostriction, and no crystalline anisotropy [4]. These magnetic properties are critical parameters for achieving a high MI response. Therefore, an essential consideration in biosensor design is the magnetic material and its thickness. Thickness variation affects material magnetization; defining the magnetization axis required to obtain a high MI response requires increasing the thickness [17]. Wicaksono et al. reported a decrease in the MI response by increasing the Cu film thickness, not the NiFe thickness, in an electrodeposited multilayered [NiFe/Cu]_N_ film [18]. However, increasing the thickness of other magnetic materials, such as amorphous materials, diminishes the magnetic softness of the material and consequently reduces the MI performance. Ideally, maximizing the MI effect requires carefully considering magnetic softness and layer thickness [19]. Thicknesses of tens of microns for both wires and ribbons are sufficient to achieve high MI response at a low frequency, 10–100 kHz when the skin depth matches the material thickness, whereas at higher frequencies of 1 GHz, a thickness of 10 µm is required. Amorphous wires and glass-coated materials have shown an excellent MI effect of 600% [20,21]. However, constructing an MI biosensor based on Py soft magnetic materials requires thinner films to achieve a similar MI response [22,23].

Biosensor geometry plays a critical role in the response of an MI biosensor. A sandwich layout displays a high MI response based on Py, amorphous ribbon, and wire materials [24,25]. However, these materials must undergo a complicated process to construct a proper biosensor geometry for a particular application. In biosensing, meander thin-film geometry with a known number of turns is ideal compared to circular wire, rectangular single line, ellipsoid, and elongated strips [21,26,27,28,29]. Sandwich structures, comprising a double magnetic layer deposited on either side of a conductive layer (non-magnetic), enhance MI in thin films [5,26,30,31,32]. Hika et al. reported a significant increase in the MI effect up to 8% when the sandwich film was incorporated in the development method compared to a single-layer film [30]. Jiang et al. have also reported an increase in the MI response from 207% in FeNi/Cu/FeNi films with a linear structure to 1247% in the same film with a horizontal meander structure with a 500 nm gap between each meander line [33]. Moreover, the largest MI ratio for sandwich structure biosensors where a wire-shaped geometry was employed is 800% [34]. However, wire-shaped geometry has limited biosensing applications and requires more complex fabrication and measurement processes than other geometries. To overcome such limitations, in this study, we develop and investigate a meander thin-film geometry with an appropriate line width and thickness ratio to construct MI biosensors achieving a high MI response.

Sandwich structures are usually patterned as rectangular stripes or ellipsoids. These patterns should be adjustable for the purposes of optimization to increase MI response. However, improper optimization diminishes MI response, as their lateral dimensions are fixed. Instead, meander line patterns are adjustable to match their later dimensions to further enhance the MI effect evidenced in Py and ferromagnetic materials [35]. Meander line geometry in the sandwich layout has shown excellent performance over the past few decades, exhibiting a higher MI effect [36]. The meander line pattern is the best adjustable shape to provide a large MI response. Moreover, meander line patterning is adaptable and straightforward to fit any measuring circuits to maximize the MI response [37]. An MI effect of 183% was achieved in a 3-turn meander line shape [29]. However, the MI response was enhanced to 190% when the lateral dimensions of the meander line were adjusted [38]. Wang et al. reported an 8.6% increase in the MI response when the turns taken by the meander line increased from 3- to 6-turns [29]. However, it is still unclear whether MI enhancement is achieved by increasing the number of meander turns or by adjusting the lateral dimensions of the meander lines. Thus, both aspects should be considered when investigating the MI effect.

The MI effect depends considerably on the lateral dimensions of the MI biosensor pattern, the number of turns, and the size of the sensing area where improper downscaling alters its magnetic properties. MI biosensors with an elongated shape are challenging to adjust compared to meander line MI biosensor shapes to enhance the MI response [39]. For example, the ellipsoid lateral dimension must be adjusted, which involves complex fabrication steps in order to correct the transverse anisotropy required to enhance the MI response [28]. Subsequently, flux leakage increases in MI biosensors with an elongated shape, as the lateral size of the biosensor is corrected [40]. Therefore, optimizing fabrication parameters is critical to enhancing the MI response.

In this study, we designed and fabricated a MI biosensor based on different sensing area sizes, thicknesses, numbers of turns to investigate their effects on the MI response. Meander-shaped MI biosensors with 3-, 6-, and 9-turns were employed in a sandwich structure with different sensing area sizes fabricated using the micro-electromechanical system (MEMS) process, including-beam evaporation and photoetching electroplating and other technical steps. The MI response of these sandwich meander-shaped MI biosensors was studied in different structural conditions. The results indicated that both the sensing area size and thickness had a significant influence on the MI response. We further analyzed and discussed the relationship between the number of turns, thickness, and the sensing area size of the meander lines.

## 2. Materials and Methods

### 2.1. MI Biosensor Principles and Physical Background

The MI effect describes the electrical impedance change in magnetic materials when subjected to an external magnetic field. In this study, nickel–iron (NiFe) was used as magnetic material, and copper (Cu) served as a conductive material for biosensor construction. MI effect variation mainly depends on the skin effect, which is the penetration of the electromagnetic field within the Cu conductive material or Cu surface when an alternating current (AC) flows through it [41]. The penetration depth δ defines the decrease of the amplitude of the electromagnetic field from the Cu surface, and it is expressed by Equation (1) [4]:

(1) $$\delta =\frac{1\;}{\sqrt{\pi f\sigma \mu }}\;$$

where *f* defines the AC frequency, and *σ* and *μ* indicate the NiFe conductivity and permeability, respectively. In practice, the *δ* defines the biosensor cross-section. When the biosensor is subjected to the external magnetic field and the AC flows through it, the employed field modifies the permeability of the biosensor material, resulting in an *MI* effect [4]. Hence, the *MI* effect depends considerably on the permeability that is controlled by adjusting the biosensor geometry. Geometry includes shapes such as meander line shape, thickness, and the number of turns. Here, we optimized these structural parameters to achieve a highly sensitive *MI* biosensor. The *MI* effect of a magnetic material is usually expressed as the relative change of impedance given by Equation (2) and is expressed in *Z*_min_ [4]. It is the minimum measured impedance when the NiFe magnetic material is exposed to a magnetic field:

(2) $$MI\left(\%\right)=\frac{Z-Z\min\;}{Z\min}\times 100\;$$

*Z*_min_ is the minimum impedance during biosensor measurements when the magnetic field strength magnetically saturates the material. In this work, the impedance is measured when the magnetic field is off, i.e., *Z*_min_ = *Z* (*H_e_* = 0). When the value of *Z* is at its maximum, the *MI* is at its maximum. This occurs when the magnetic field intensity “*H_e_*” saturates the NiFe material, resulting in maximum permeability and minimum skin depth. Hence, we used a permanent magnet to generate a constant and strong magnetic field to maintain high permeability.

A critical factor in biosensor design is the *MI* response and its relationship to the magnetic field and sensor geometry. The maximum *MI* response occurs when *Z*(*H*) exhibits a maximum change as anisotropy affects it. The maximum *MI* response can be achieved by longitudinal anisotropy, which occurs when the *MI* biosensor is placed parallel to the applied external magnetic field and the current flow. Transverse anisotropy arises when the *MI* biosensor is perpendicularly positioned to the applied external magnetic field and the current flow. For the longitudinal anisotropy configuration, at *H_e_* = 0, the transverse permeability is maximum. For transverse anisotropy, the transverse permeability is at its maximum at H^k^, which is given by Equation (3) [42]:

(3) $$H^{k}=\frac{2K}{\mu_{0}\;Ms\;},\;$$

where *K* indicates the anisotropy constant, and *M_s_* defines saturation magnetization. The maximum *MI* response is achieved when H^k^ is at its minimum.

### 2.2. MI Biosensor Design and Principles

**Figure 1:**
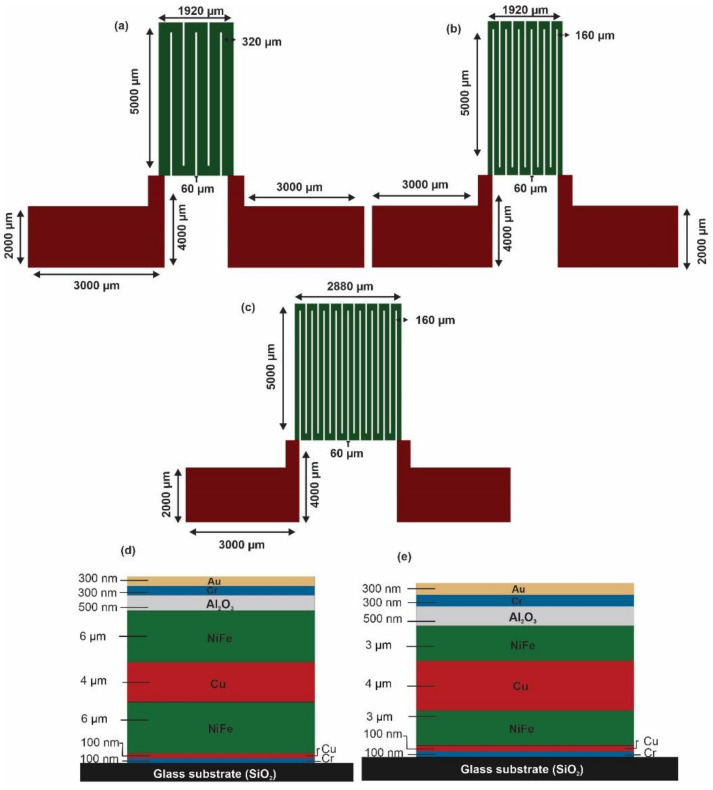
MI biosensor design and geometry. (**a**) The geometry of the 3-turn meander line MI biosensor. (**b**) The geometry of the 6-turn meander line MI biosensor. (**c**) The geometry of the 9-turn meander line MI biosensor. (**d**) MI biosensor materials with 6 μm thick magnetic materials. (**e**) MI biosensor materials with 3 μm thick magnetic materials.

A magneto-impedance biosensor was designed and constructed using NiFe soft magnetic material. It was designed and patterned into a meander structure with a length of 5 mm and with various line widths and a gap of 60 µm between each line (Figure 1). Three configurations were accomplished based on the number of turns (3-, 6- and 9-turns), as each “n” shape in the meander structures represented one turn. The width of each meander line was 320 µm in the 3-turn configuration and 160 µm in the 6- and 9-turn configurations. The line width in the 9-turn MI biosensor configuration was identical to the 6-turn MI biosensor line width of 160 µm. Figure 1a depicts the 3-turn MI biosensor configuration, where the meander line is 160 µm wider than the 6-, and 9-turn MI biosensors structures in order to achieve a sensing area size equal to the 6-turn MI biosensor sensing area size. Figure 1b shows the schematic of the 6-turn MI biosensor design, which is identical to the 9-turns design in Figure 1c with the exception of the number of turns. The 9-turn MI biosensor was designed to provide the largest sensing area size of 5 mm × 2.88 mm, where an equal sensing area size of 5 mm × 1.92 mm resembled the 6- and 3-turn MI biosensors configurations. The sensing area size variations were designed to investigate the relationship between the number of turns and the influence of the size of the sensing area on the MI effect and to determine an optimal sensing area for the turns ratio, which is required for a high MI response. All three MI biosensor configurations have identical contact pads (electrode) made of copper (Cu), which are marked in red as shown in Figure 1. Green resembles the soft magnetic material (NiFe).

A meander thin-film MI biosensor was constructed with double magnetic layers based on two different thicknesses to investigate the effect of MI when it is based on thickness variation and to determine an optimal thickness to sensing area size ratio to achieve maximum MI response. Figure 1d,e display the cross-section views of the MI biosensor materials and layers where the magnetic layer (NiFe) thickness was 6 µm and 3 µm, respectively. A thickness of 4 µm Cu was used as a conductive layer in all configurations. The Cu conductive layer has a similar shape to the NiFe magnetic layer with the exception of the line width of 120 µm and the extended electrode fabricated into a rectangle with a length of 4000 μm and a width of 3000 μm. All of the other materials layers were identical to the NiFe magnetic material shape and comprised of 100 nm chromium/copper (Cr/Cu) as a seed layer, 500 nm aluminum oxide (Al_2_O_3_) as an insulation layer, and 300 nm chromium/gold (Cr/Au) to be used for potential biosensor functionalization. The NiFe magnetic layer was processed to the length of 5000 μm in all configurations, a width of 160 μm in the 6- and 9-turns configurations, a width of 320 µm in the 3-turn configuration, and a thickness of 3 and 6 μm in all of the MI biosensors. The Cu conductive layer had a similar length of 5000 µm, a width of 120 µm, and a thickness of 4 µm.

The overall MI biosensor design consisted of Cr/Cu/NiFe/Cu/NiFe/Al_2_O_3_/Cr/Au films, as shown in Figure 1d,e. The MI biosensor sensing element was made of NiFe/Cu/NiFe films, as NiFe films exhibit large permeability, low magnetostriction, and low crystalline anisotropy. The Cu film served as a conductive layer enclosed by the NiFe films to obtain a closed magnetic flux path to enhance the MI response [4]. An Au film was employed as a biomolecular immuno-platform for a potential sandwich antibody immunoassay. The Al_2_O_3_ film served as an insulation layer and was inserted between the sensing element and the immuno-platform.

### 2.3. MI Biosensor Fabrication

**Figure 2:**
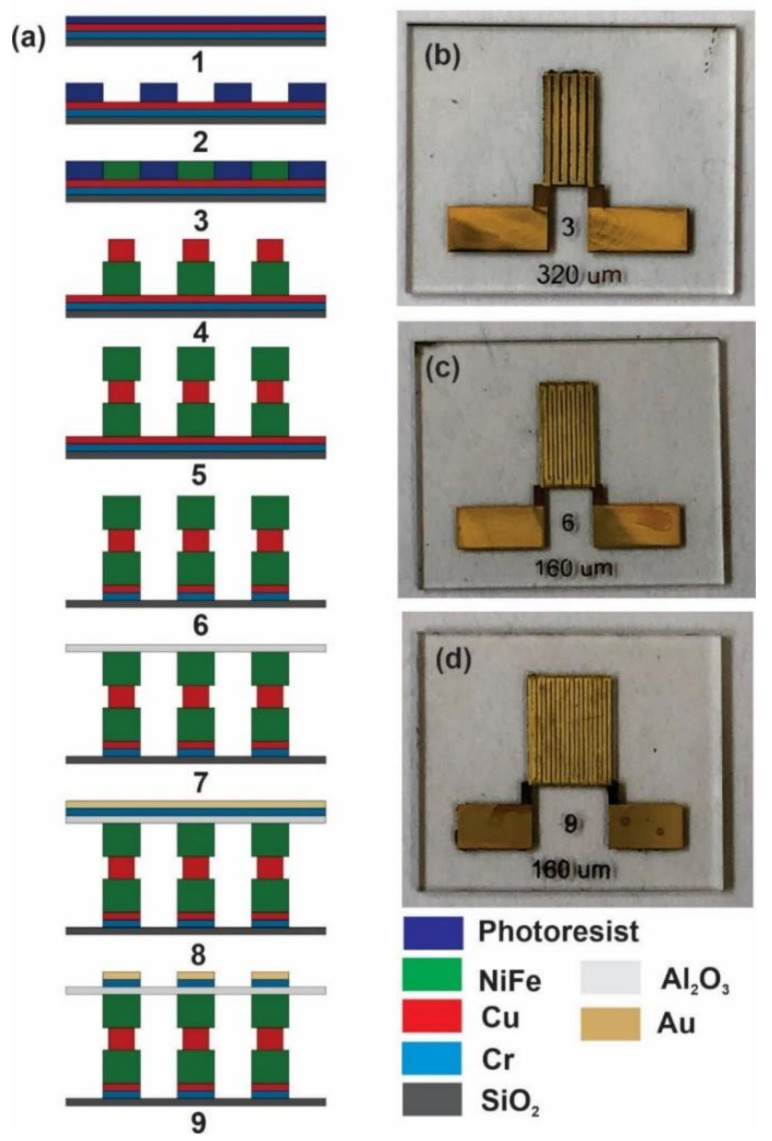
The fabrication process of the MI biosensor. (**a**) The preparation process of the meander thin-film MI biosensor. (**b**) A photograph of the 3-turn biosensor (turn width 320 μm). (**c**) A photograph of the 6-turn biosensor (turn width 160 μm). (**d**) A photograph of the 9-turn biosensor (turn width 160 μm).

The MI biosensor was fabricated by MEMS technology, and the preparation process is shown in Figure 2a. First, a seed layer of Cr/Cu of 100 nm was deposited on a glass substrate by means of electron-beam evaporation followed by the spin-coating of 10 µm photoresist (Figure 1a, step 1). Second, the photoresist was photoetched and patterned into a meander shape (Figure 1a, step 2). Then, the bottom NiFe magnetic layer was electrodeposited on the seed layer using the electroplating condition provided in Table 1 with various thicknesses: 3 µm and 6 µm (Figure 1a, step 3). Then, the Cu conductive layer was electrodeposited using the electroplating conditions shown in Table 2 and demonstrated a 4 µm thick (Figure 1a, step 4). The top NiFe magnetic layer was then electrodeposited with the same electroplating conditions in Table 1, which was identical thickness to the bottom NiFe layer (Figure 1a, step 5). Then, the Cr/Cu seed layer was removed using the reactive ion etching (RIE) technique (Figure 1a, step 6). An insulation layer of Al_2_O_3_ that was 300 nm thick was then deposited by means of electron-beam evaporation followed by an etching process to bare the Cu electrode (Figure 1a, step 7). Finally, a Cr/Au that was 300 nm thick was deposited as an immuno-platform and functionalization layer on the insulation layer, and it was then etched into the flexural meander structure (Figure 1a, step 8, 9). This step concluded the MI biosensor fabrication process, and three MI biosensor configurations were achieved (Figure 2b–d).

A critical parameter in MI biosensor fabrication and measurement is the external magnetic field used to define the anisotropy during fabrication and to magnetize the magnetic sensing element during testing. A constant magnetic field H_k_ was induced by a permanent magnet along the transverse direction during the electroplating fabrication process to create anisotropy. Another permanent magnet was used to generate an external magnetic field *H_e_* that is required for the measurements. A higher longitudinal MI response is more likely to be obtained when H_k_ = *H*_e_, where the permanent magnet can be placed opposite the MI biosensor meander lines according to Equation (3) [4]. The layer thickness was measured by a surface profiler (SP).

### 2.4. Experimental Setup and Measurement

**Figure 3:**
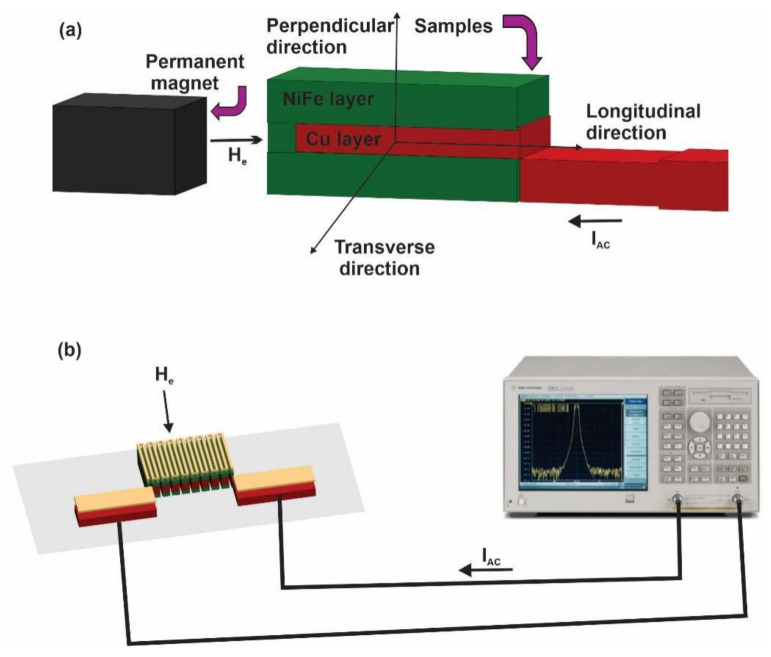
The experimental process and setup. (**a**) The magnetic field orientation; (**b**) the experimental setup.

The magnetic domain is defined along the meander length and width and is perpendicular to the meander plane. It resembles the longitudinal, transverse, and perpendicular MI response, as shown in Figure 3a. The longitudinal MI ratio is presented in this measurement. A permanent magnet with a length of 70 mm and a thickness of 20 mm was used to generate an external magnetic field to magnetize the biosensor sensing element during the measurements. It was placed in the longitudinal direction in front of the MI biosensor, resulting in a longitudinal MI effect. The MI biosensor was securely fixed on a probe station parallel to the permanent magnet’s central axis and the longitudinal external field *H*_e_, as shown in Figure 3a. The permanent magnet generated a 0–20 Oe magnetic field during testing by adjusting the distance between the MI biosensor and the permanent magnet. The MI measurements were conducted using an R&S^®^ ZVL vector network analyzer with two contact terminals. The AC flowed through the MI biosensors with a sweep frequency range of 1–40 MHz and a constant current amplitude of 10 mA, as shown in Figure 3b. The relative change in impedance indicates the magnitude of the MI response, which is denoted as the MI ratio. It was calculated from the *Z*(*H*) curves and was defined by Equation (4) [38]:

(4) $$MI\left(\%\right)=100\%\;\times \;\frac{Z\left(H\right)-Z\left(H_{0}\right)\;}{Z\left(H_{0}\right)}\;$$

*Z*(*H*) is the magneto-impedance when the longitudinal magnetic field *H_e_* is ON, and *Z*(*H*_0_) is the magneto-impedance when the magnetic field is OFF. The *Z*(*H*) is obtained by the equation for impedance, Equation (5) [38]:

(5) Z=R+jωL

*Z* describes the impedance, *R* denotes the resistance, the real part of the impedance, and *L* represents the inductance, the imaginary part of the impedance.

### 2.5. Statistical Analysis

We used Microsoft Excel to convert the real and imaginary phase data into impedance (*Z*). The relative change in *Z* indicates the magnitude of the MI response, which is denoted as the MI ratio, and it is calculated from the *Z*(*H*) curves when the external magnetic field is applied. GraphPad Prism 7.0 software and Microsoft Excel were used to perform statistical analysis and to create plots. Optimization of the sensing area size and thickness was performed using the MI response values obtained from the biosensor. This represents the electrical impedance changes of the sensing element measured when the magnetic field was ON and OFF. The 3-, 6- and 9-turn MI biosensors were used for testing, and five measurements for each biosensor type were conducted. Average MI ratio values were derived and compared between the three biosensor types using a one-way ANOVA test. Relative standard deviation (RSD) was calculated using Microsoft Excel. The resultant sensing area size from the biosensor turns, and the different film thicknesses were compared. The optimal sensing area size and thickness was chosen based on the largest MI response and the biosensor with the least amount of variation. A bar chart was generated, and *p*-values ≤ 0.05 were considered statistically significant.

## 3. Results and Discussion

The MI biosensors were constructed based on fabrication parameters that included various thicknesses, sensing area sizes, and numbers of turns to investigate the impact of these parameters on the MI response. The results are presented and follow the impact of the fabrication parameters, including the number of turns taken by the film, film thickness, and size of the film sensing area. Moreover, the MI biosensor reproducibility and stability results are presented.

### 3.1. The Influence of Number of Turns Taken by the Film and the Film Thickness on the MI Response

#### 3.1.1. MI Biosensor Configuration—9 Turns

**Figure 4:**
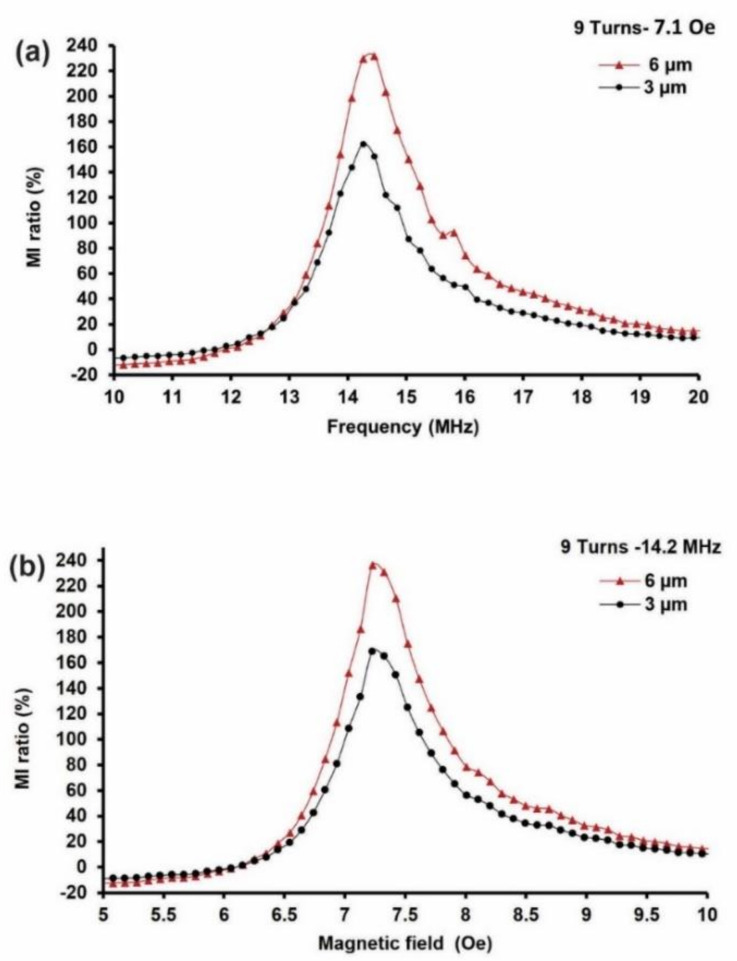
Results of the 9-turn MI biosensor based on different film thicknesses. (**a**) Frequency dependence of the MI ratio at 7.1 Oe. (**b**) Field dependence of the MI ratio obtained at 14.2 MHz.

The relationships between the number of turns and the film thickness for each MI biosensor are presented in this section. The results of the 9-turn MI biosensor based on 3-µm and 6 µm film thicknesses are shown in Figure 4. All of the measurements were conducted at a frequency range of 1–40 MHz and at an external magnetic field, “H_e_”, of 0–20 Oe to determine the peak of the impedance change. A vector network analyzer, the R&S^®^ ZVL, was used for the MI measurements, and a permanent magnet was used to generate the external magnetic field. The MI biosensor consisted of Cr/Cu/NiFe/Cu/NiFe/Al_2_O_3_/Cr/Au films, where the NiFe/Cu/NiFe served as a sandwich magnetic material. Two thicknesses were investigated: 3 µm and 6 µm.

The results indicated that the impedance change peaked at 14.2 MHz and maximized when subjected to the applied external magnetic field at 7.1 Oe. The maximum MI effect was found at 7.1 Oe when the permanent magnet was placed 4 cm away from the MI biosensor. The MI ratio was calculated based on Equation (4), where *Z*(*H*) denotes the MI response when the magnetic field *H_e_* was ON; the permanent magnet was placed at a 4 cm distance away from the MI biosensor, and *Z*(*H*_0_) was OFF when the permanent magnet was removed. Figure 4 presents the 9-turn MI biosensor results based on 6 µm and 3-µm film thicknesses. Figure 4a displays the MI ratio’s frequency dependence at 7.1 Oe, whereas Figure 4b shows the field dependence of the MI ratio obtained at 14.2 MHz. The results revealed a maximum MI ratio of 238% in the 6 µm thick film MI biosensor, which is 70% over the maximum MI ratio of 168% for the 3 µm thick film at 14.2 MHz and 7.1 Oe.

Here, the increase in the MI ratio can be partially attributed to the number of turns made by the meander line. The number of turns (9 turns) acts as a magnetic flux, where the magnetic flux of the adjacent sandwich NiFe film interacts with each other by the mutual inductance of the magnetic chain. Due to this mutual-inductance effect, the more turns that are made, the greater the change in the inductance ratio in the meander structure is. Hence, a more extensive change ratio can be obtained by Equation (4), leading to a greater MI response. Moreover, the skin effect, which is the tendency of the alternating current to become distributed within the Cu conductive layer such that the current density is larger on the surface of the Cu layer and decreases exponentially with greater depths in the Cu material (skin depth), has been attributed to the MI response increase. Here, the skin depth matches the NiFe/Cu/NiFe material thickness, whereas the NiFe is 6 µm thick, achieving a 70% increase. Furthermore, the sandwich structure geometry has contributed to MI enhancement in the areas where the large permeability magneto-inductive effect dominates the skin effect, allowing it to overcome the skin depth, resulting in large impedance changes. To achieve a higher MI response, we optimized structural parameters such as film thickness, the number of turns, and sandwich layout.

In this work, the MI response peaked at 238% and 168% at 14.2 MHz under 7.1 Oe in the 9-turn sample with 6 µm and 3 µm film thicknesses. This peak value is much larger than the maximum MI ratio reported by Yang et al.,where more turns (10-turns) were employed, and the peak field of 7.1 Oe is much lower than their reported peak, 17 Oe [43]. Usually, a pair or more of Helmholtz coils are commonly used to generate external magnetic fields [22,23,29,37,43,44,45,46,47,48]; they are hazardous, consume a great deal of power, are expensive, and require complex design and fabrication. Consequently, the applications MI biosensors are limited if Helmholtz coils are utilized. However, we overcame such limitations by using a cost-effective and straightforward rectangular permanent block magnet to generate the required external magnetic field to achieve a similar MI response under lower magnetic field conditions than those of reported studies [36,39].

#### 3.1.2. MI Biosensor Configuration—6 Turns

**Figure 5:**
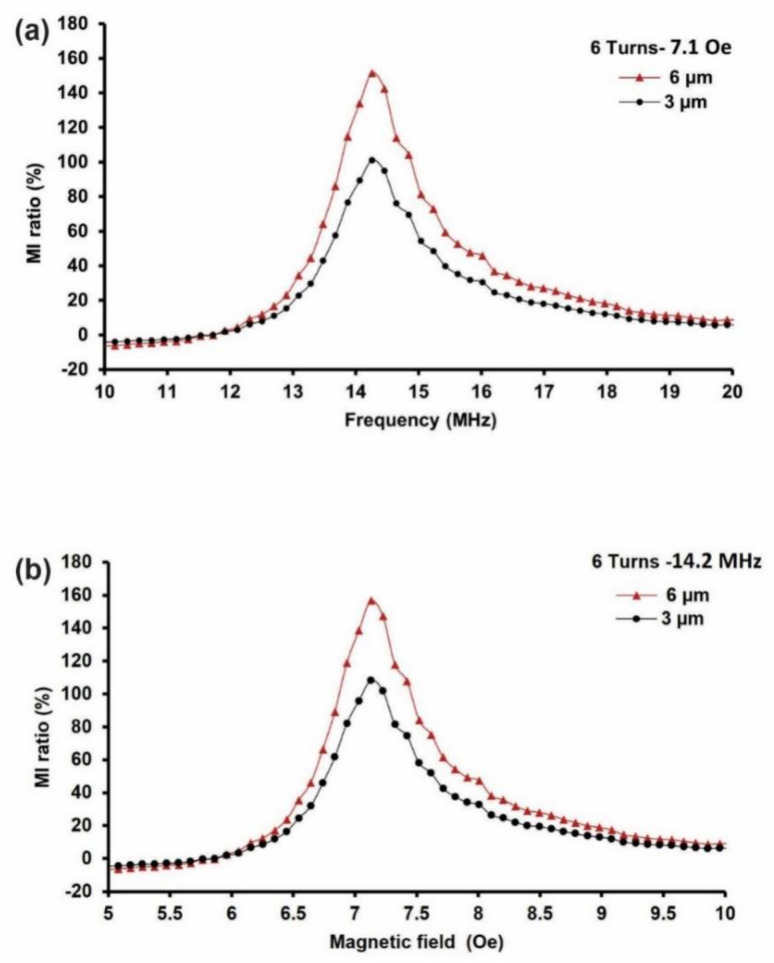
Results of the 6-turn MI biosensor based on different film thicknesses. (**a**) Frequency dependence of the MI ratio at 7.1 Oe. (**b**) Magnetic field dependence of the MI ratio obtained at 14.2 MHz.

The 6-turn MI biosensor is identical to the 9-turn MI biosensor, with the exception of the number of turns used to investigate the turn impact on the MI response based on 3 µm and 6 µm film thicknesses. The same measurement setup and conditions were used: 1–40 MHz and an external magnetic field, “*H_e_*”, of 0–20 Oe. The maximum impedance change was at 14.2 MHz under 7.1 Oe, as the permanent magnet was placed 4 cm apart from the MI biosensor. Figure 5 presents the 6turn MI biosensor results based on 6 µm and 3 µm film thicknesses. The MI ratio frequency and magnetic field dependency are shown in Figure 5 a,b. The results showed that the MI ratio increased by 49% from 108% to 157% as the thickness increased from 3 µm to 6 µm. These results concur with the 9-turn MI biosensor results, with the exception of the MI ratio magnitude, which dropped as the number of turns decreased.

It is worth noting that the number of turns taken by the film is essential to the design of an MI biosensor when trying to achieve a higher MI response. We found that the MI ratio in the 6- and 9-turn models increased as the number of turns increased at the proper thickness. For the 6-µm film thickness, the MI ratio of 238% (9-turns) was higher than the MI ratio of 157% (6-turns) by 81%. It should be noted that an increase of three turns increased the MI ratio by 81%. Similarly, for the 3 µm film thickness, the MI ratio increased by 60%, from 108% to 168%, in the 6- and 9-turn configurations. Hence, the 9-turn meander line with a 6-µm film thickness could be the ideal parameters for MI biosensor design to achieve a higher MI response. As discussed above, the increase in the MI ratio can mainly be attributed to the large magnetic flux produced by the 9-turn configuration compared to the 6-turn configuration, as the magnetic flux is proportional to the number of turns. This may differ if the size of the meander line is altered; hence, further investigation is required. Moreover, the 6-turn MI biosensor displayed an MI response that was higher than the MI response reported by Wang et al. using the same biosensor conditions [29].

#### 3.1.3. MI Biosensor Configuration—3 Turns

The 3-turn MI biosensor exhibited similar characteristics to the 6- and 9-turn MI biosensors, with the exception of the number of turns and the width of the meander line. The width of the meander line was 160 µm wider than the 60- and 9-turn configurations. The measurements were conducted using the same setup and conditions as above.

**Figure 6:**
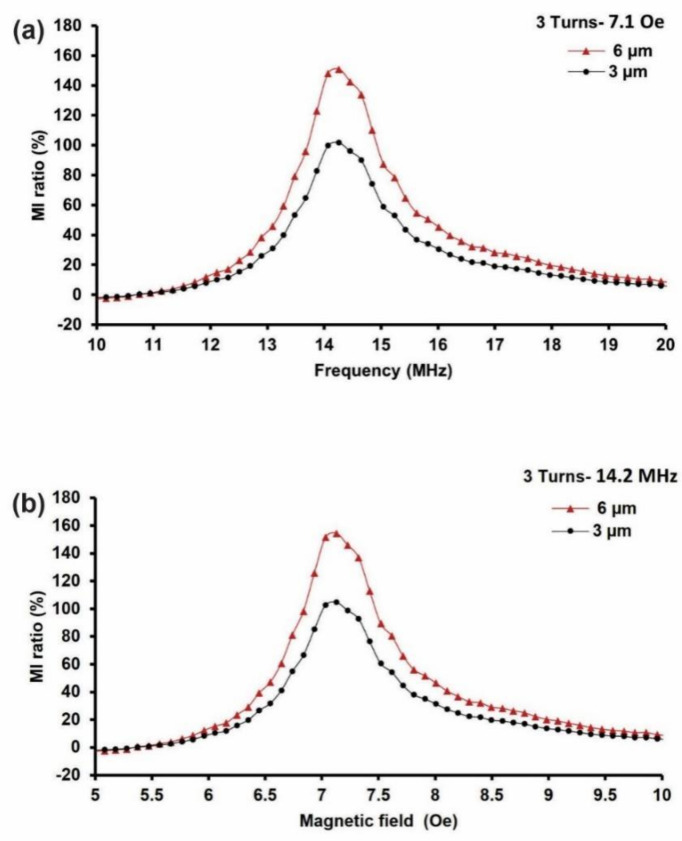
Results of the 3-turn MI biosensor based on different film thicknesses. (**a**) Frequency dependence of the MI ratio at 7.1 Oe. (**b**) Magnetic field dependence of the MI ratio obtained at 14.2 MHz.

Figure 6 depicts the 3-turn MI biosensor results based on the frequency and magnetic field dependency. The results indicated that the maximum MI ratio increased from 105% to 155% as the film thickness increased from 3 µm to 6 µm at 14.2 MHz and 7.1 Oe. The MI ratio increased by 50%, a value that is almost the same as the 49% seen for the 6-turn MI biosensor. However, the 9-turn MI biosensor displayed the largest MI ratio increase by 70% as the thickness increased from 3 µm to 6 µm under the same conditions. It was shown that increasing the 6-turns MI biosensor by three turns increased the MI ratio by 81%. Contrarily, decreasing the 6-turn MI biosensor configuration by three turns (3-turn MI biosensor) did not affect the MI ratio. The 3-turn MI biosensor showed similar results to the 6-turn MI biosensor despite discrepancies in the turns. Table 3 compares and lists the MI response of the 3-, 6- and 9- turn MI biosensors based on the 3 µm and 6 µm thicknesses and the sensing area size.

The 3- and 6-turn MI biosensors were expected to provide different MI ratios because when there are more turns, the MI ratio is higher, especially when compared to the configuration results of the 6-turn and 9-turn models. This discrepancy could be attributed to the meander line geometry of the 3-turn MI biosensor possess. Hence, further investigation is required to understand whether the MI ratio increase was mainly dependent on the number of turns taken by the meander line or the width of the turns (more details in the following sections). Still, the maximum MI ratio, 155%, in the 3-turn MI biosensor was comparable to reported MI ratios [38,47,49].

### 3.2. The Influence of Film Sensing Area Size on the MI Response

An optimal ratio of the film sensing area size and the number of turns is critical to the design of highly sensitive MI biosensors. Here, three sensing area sizes were designed: 5 mm × 2.88 mm, 5 mm × 1.92 mm, and 5 mm × 1.92 mm. The sensing area size was determined by the number of turns taken by the meander line (3, 6, and 9) and the line width, which was 160 µm wide in the 9- and 6-turn configurations and was 320 µm wide in the 3-turn MI biosensors. The resultant sensing area sizes were 14.4 mm^2^ in the 9-turn configuration and 9.6 mm^2^ in the 6- and 3-turn configurations. The 9-turn MI biosensor contained the largest sensing area size, whereas the 6- and 3-turn MI biosensors exhibited a similar sensing area size despite the differences in the number of line turns because the line width in the 3-turn biosensor is doubled. Sensing area size variation was employed to investigate its influence on the MI effect and to determine the ratio of the optimal sensing area size to the number of turns required to enhance the MI response. Moreover, the intention was also to investigate whether the MI ratio is greatly affected by the sensing area size or the number of turns, as partially shown in the previous sections.

**Figure 7:**
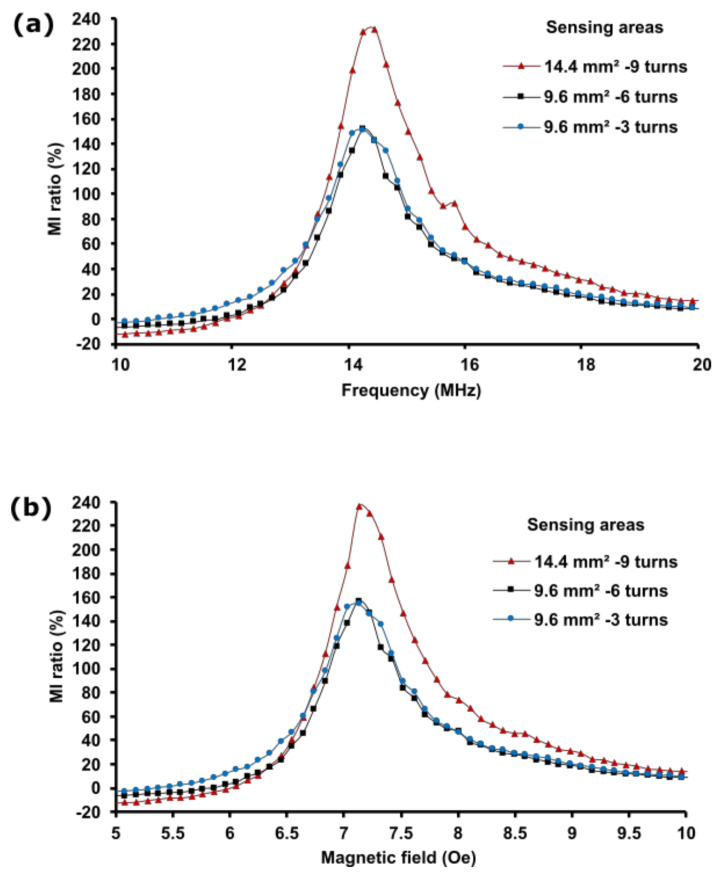
MI ratio based on sensing area variation. (**a**) Frequency dependence of the MI ratio. (**b**) Field dependence of the MI ratio.

Figure 7 shows the plots for the impedance change ratio of the MI biosensor(%MI), which are based on the frequency and magnetic field. Figure 7a depicts the frequency dependence of the MI ratio on various magnetic film sensing areas, whereas Figure 7b shows the magnetic field dependence of the MI ratio. The impedance was measured at 14.2 MHz when subjected to an external magnetic field of 7.1 Oe applied along the longitudinal direction of the MI biosensor. The maximum MI was obtained at the magnetic field of 7.1 Oe, which was almost equal to the magnitude of the field required to saturate the magnetization of the NiFe magnetic films along the longitudinal direction to investigate the permeability. The results showed that the 14.4 mm^2^ sensing area yielded the maximum MI ratio, as the biosensor comprises nine turns. The 6- and 3-turn MI biosensors exhibited almost similar MI ratios, as they possess a similar sensing area size of 9.6 mm^2^. The maximum MI ratios based on the sensing area sizes, 14.4 mm^2^, 9.6 mm^2^ and 9.6 mm^2^, were 238%, 157%, and 155%, representing the 9-, 6- and 3-turn MI biosensors, respectively. Thus, the relationship between the number of turns and the MI ratio is not proportional. We noted a linear relationship between the MI ratio and the film sensing area size; the MI ratio increased as the film sensing area size increased.

The MI response variation could be attributed to the permeability of the magnetic films affected by the meander line size. At 14.2 MHz, the permeability of the film sensing area (14.4 mm^2^) improved compared to the permeability of the 9.6 mm^2^ film sensing area. This could be attributed to the reduction in the eddy current loss controlled by the shape and the size of the film sensing area rather than the number of turns. It is worth noting that the eddy current loss in the 14.4 mm^2^ sensing area shape is much less than it is in the 9.6 mm^2^ sensing area biosensors. This explains why the permeability retains its value at 14.2 MHz, achieving a high MI response. Based on these results, a 14.4 mm^2^ sensing area size comprising a 160 µm and 5 mm meander line width and length, 9-turns, and 6 µm film thickness are the preferred parameters for designing highly sensitive MI biosensors. These findings are congruent with the MI ratio increase reported by Arribas et al., where they linked the increase in the MI ratio to the permeability changes based on different film sensing areas sizes despite using a single magnetic layer sensor [49]. However, the eddy current investigation and its impact on the permeability was absent. A theoretical investigation is required to understand the mechanism of the magnetization process concerning the eddy current, permeability, and its impact when the sensing area size varies.

### 3.3. MI Biosensor Comparison and Reproducibility

MI ratio variation was attributed to the variation in the size of the biosensor sensing areas rather than the number of turns due to the permeability change of the magnetic films affected by the meander line size, as shown in the previous section (Figure 7). It was also found that the larger the sensing area size, the stronger the generated magnetic field and the higher the permeability. Thus, it is critical to compare and investigate whether magnetic properties are retained or changed when more tests are performed. A comparison between biosensor types was performed using a one-way ANOVA (multiple comparisons) test to determine and confirm the optimal sensing area size and sensor type based on the MI values obtained from the biosensors. Relative standard deviation (RSD) was calculated using Microsoft Excel.

**Figure 8:**
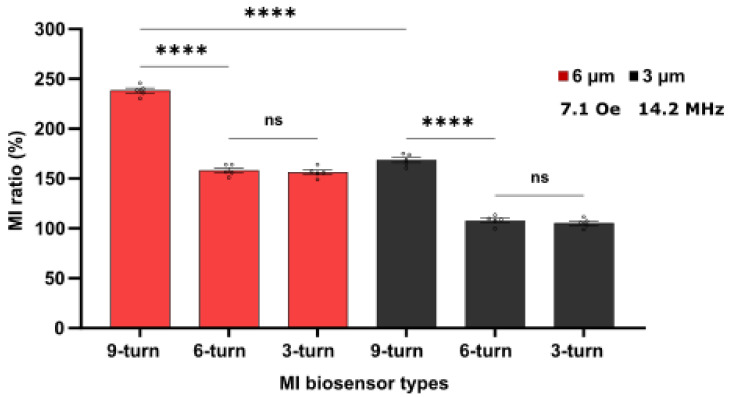
Comparison of the MI biosensor types (3-, 6-, 9-turn) with 6 µm thickness (red bar) and 3 µm thickness (black bar). The dot with the error bar represents the mean and standard error of the mean (±SEM), *n* = 5. The statistical significance is based on the ordinary one-way ANOVA (multiple comparisons) whereas *p* = 0.0605 (non-significant, ns), and <0.0001 (****).

Figure 8 shows the MI ratios of fives measurements for each MI biosensor type with sensing areas sizes of 14.4 mm^2^ (9-turns), 9.6 mm^2^ (6-turns), and 9.6 mm^2^ (3-turns) with 3-µm and 6-µm film thicknesses (black bar and red bar, respectively). Using one-way ANOVA compassion, the %MI was not significantly different between the 3- and 6-turns MI biosensor types (*p* = 0.06075). Nonetheless, the 9-turn MI biosensor (6 µm thick) was significantly different and displayed the largest MI ratio compared to the 6- and 3-turn MI biosensors. Moreover, the 9-turn MI biosensor (3 µm thick) also showed a larger MI ratio than the 3- and 6-turn MI biosensors. The 3- and 6-turn MI biosensors showed similar trend variation in the MI ratio, which may be related to the similar sensing area size variations. Based on the calculated RSD values, the 9-turn MI biosensor (14.4 mm^2^ sensing area size) in 6 µm film thickness preserved its magnetic properties and showed excellent reproducibility with an RSD of 2.12%, indicating good precision compared to the RSD of 3.22% of the same sensor with a 3 µm film thickness. In the 6- and 3-turn MI biosensors (9.6 mm^2^ sensing area), the RSD increased to 3.14% and 3.10% for the 6 µm film thickness and to 4.12% and 4.04% for the 3 µm film thickness. Both the 6- and 3-turn MI biosensors displayed almost similar RSD, as they exhibited similar MI ratios. Therefore, excellent reproducibility is retained in the 9-turn biosensors.

MI biosensor geometry plays a vital role in magnetic signal detection. This work optimized structural parameters to achieve highly sensitive biosensors based on biosensor sensing area size, thickness, and the number of turn variations. Based on these findings, the 9-turn MI biosensor could be suitable for biomagnetic detection applications. The 9-turn MI biosensor’s (14.4 mm^2^) excellent stability was attributed to the appropriate ratio of the film thickness (6 µm) and the sensing area size (14.4 mm^2^), resulting in the stability and excellent superparamagnetism of the NiFe film. Here, the NiFe film thickness directly impacts the skin effect, where the permeability increases as the penetration depth decreases. The penetration depth matched the material thickness at 14.2 MHz, producing significant impedance variations. The excellent reported precision of the 9-turn MI biosensor supports the reliability of measurement results, making it a suitable biosensor for medical applications compared to other reported low MI biosensor responses. For example, MI biosensors have been reported to detect the cancer biomarker alpha-fetoprotein (AFP) with an MI ratio of 55% to 70% [29,50], the cardiovascular disease and inflammations biomarker C-reactive protein (CRP) with an MI ratio of 100% [48], the prostate-specific antigen (PSA) with an MI ratio of 95% [51], and protein A with an MI ratio of 115% [46].

## 4. Conclusions

We have investigated structural parameters of biosensors that are able to influence the MI effect. We fabricated an MI biosensors-based sandwich NiFe/Cu/NiFe film with a meander configuration by means MEMS technology into 3-, 6-, and 9-turn configurations with different film thicknesses prepared using electroplating technology. The structural parameters, including film thickness, sensing area size, and the number of turns, and how they influence the MI effect, have been thoroughly investigated. We speculated an ideal NiFe film thickness and sensing area size ratio to obtain the maximum MI ratio. We predicted that the MI ratio significantly depends on the sensing area size rather than the number of turns by designing 3- and 6-turn models with equal sensing area sizes to investigate this hypothesis. The MI ratio results showed a variation trend from increasing to decreasing with the increase of the film sensing area size and the thickness rather than the number of turns. The maximum MI ratio obtained by the 9-turn layout was 80% larger than the 3- and 6-turn structures at 14.2 MHz and 7.1 Oe. The NiFe film thickness of 6 µm with a sensing area size of 14.4 mm^2^, which resembled a 9-turn MI biosensor, is the optimum ratio yielding the maximum MI ratio. We found that the 3- and 6-turn MI biosensors shared a common characteristic: the MI ratio peaked at an almost similar value at the 3-µm and 6 µm film thicknesses. Thus, the relationship between the number of turns and the MI ratio is not proportional. We noted a linear relationship between the MI ratio and the film sensing area size; the MI ratio increased as the film sensing area size increased. It is worth noting that the magnetic peak field increased with the increase of the sensing area size and thickness, and the MI ratio in the 3-, 6- and 9-turn biosensors reached the maximum at the same magnetic field intensity indicating good stability and reproducibility. Excellent stability and reproductivity were retained in the 9-turn MI biosensors, as indicated by the low RSD of 2.12%, making its structural parameters ideal for biosensor design. These findings provide valuable information for the design of highly sensitive MI biosensors to detect weak biomagnetic signals in medical applications. However, other parameters, such as Cu conductive layer thickness, its line dimension, and the gap between each meander line, may also impact the MI response and should be investigated in future studies.

## Figures and Tables

**Table 1:** Electroplating conditions for soft magnetic layers (Ni_82_Fe_18_).

Composition	Content
Nickel (II) sulfate (NiSO_4_·6H_2_O)	200 g/L
Iron (II) sulfate (FeSO_4_·7H_2_O)	8 g L^−1^
Nickel (II) chloride (NiCl_2_·6H_2_O)	5 g L^−1^
Boric acid (H_3_BO_3_)	25 g L^−1^
Saccharin (C_7_H_5_NO_3_S)	3 g L^−1^
PH	2.5–3.0
Temperature	20–30 °C

**Table 2:** Electroplating conditions for the conductive layer (Cu).

Composition	Content
Copper (II) sulphate (CuSO_4_)	350 g L^−1^
Sulfuric acid (H_2_SO_4)_	30 mL L^−1^
Chloride acidic PEG	5 g L^−1^
PH	2.5–3.0
Temperature	20–30 °C

**Table 3:** MI response based on film thickness, sensing area size expressed in mm^2^, and number of turns taken by the film.

Film Thickness	MI Response		
	3-Turn (9.6 mm^2^)	6-Turn (9.6 mm^2^)	9-Turn (14.4 mm^2^)
3 µm	105%	108%	168%
6 µm	155%	157%	238%
